# Isoalantolactone Suppresses Glycolysis and Resensitizes Cisplatin-Based Chemotherapy in Cisplatin-Resistant Ovarian Cancer Cells

**DOI:** 10.3390/ijms241512397

**Published:** 2023-08-03

**Authors:** Jaemoo Chun

**Affiliations:** KM Convergence Research Division, Korea Institute of Oriental Medicine, Daejeon 34054, Republic of Korea; jchun@kiom.re.kr; Tel.: +82-42-868-9511

**Keywords:** ovarian cancer, cisplatin resistance, cancer metabolism, glycolysis, isoalantolactone, combination therapy

## Abstract

Cisplatin is a potent chemotherapeutic drug for ovarian cancer (OC) treatment. However, its efficacy is significantly limited due to the development of cisplatin resistance. Although the acquisition of cisplatin resistance is a complex process involving various molecular alterations within cancer cells, the increased reliance of cisplatin-resistant cells on glycolysis has gained increasing attention. Isoalantolactone, a sesquiterpene lactone isolated from *Inula helenium* L., possesses various pharmacological properties, including anticancer activity. In this study, isoalantolactone was investigated as a potential glycolysis inhibitor to overcome cisplatin resistance in OC. Isoalantolactone effectively targeted key glycolytic enzymes (e.g., lactate dehydrogenase A, phosphofructokinase liver type, and hexokinase 2), reducing glucose consumption and lactate production in cisplatin-resistant OC cells (specifically A2780 and SNU-8). Importantly, it also sensitized these cells to cisplatin-induced apoptosis. Isoalantolactone–cisplatin treatment regulated mitogen-activated protein kinase and AKT pathways more effectively in cisplatin-resistant cells than individual treatments. In vivo studies using cisplatin-sensitive and resistant OC xenograft models revealed that isoalantolactone, either alone or in combination with cisplatin, significantly suppressed tumor growth in cisplatin-resistant tumors. These findings highlight the potential of isoalantolactone as a novel glycolysis inhibitor for treating cisplatin-resistant OC. By targeting the dysregulated glycolytic pathway, isoalantolactone offers a promising approach to overcoming drug resistance and enhancing the efficacy of cisplatin-based therapies.

## 1. Introduction

Ovarian cancer (OC) constitutes a significant global health concern, as it represents one of the leading causes of cancer-related deaths among women [1]. The standard treatment procedure for OC includes surgery followed by chemotherapy [2]. Cisplatin, along with other drugs, is considered an effective first-line chemotherapy option [3]. As a platinum-based chemotherapeutic agent, cisplatin has demonstrated remarkable efficacy in cancer management [4]. However, despite the favorable initial response, most patients develop resistance to cisplatin after repeated exposure [5]. Overcoming drug resistance is crucial for improving patient outcomes and increasing the effectiveness of existing therapies.

The acquisition of cisplatin resistance is a complex process involving various molecular alterations within cancer cells [6]. With an increasing understanding of the mechanisms by which cancer cells develop chemoresistance, various mechanisms have been implicated in this process, including variations in drug metabolism, mutations in drug targets, changes in DNA synthesis and repair, the formation of cancer stem cells, immunosuppression, the inactivation of apoptotic genes, and the activation of anti-apoptotic genes [7,8]. Among these mechanisms, alterations in cellular metabolism have gained increasing attention [9]. One of the prominent metabolic adaptations observed in cancer cells is the upregulation of glycolysis, a metabolic pathway that generates energy and biosynthetic precursors for rapid cell proliferation [10]. This metabolic shift, known as the Warburg effect, allows cancer cells to thrive in the hypoxic and nutrient-limited tumor microenvironment [11]. Concerning cisplatin resistance, the dysregulated glycolytic pathway provides a survival advantage to resistant cancer cells. Consequently, targeting the dysregulated glycolytic pathway presents a promising strategy for overcoming cisplatin resistance in cancer [12,13]. Given that inhibiting glycolysis may disrupt the metabolic adaptations that enable cancer cells to survive and proliferate in the presence of cisplatin, identifying effective glycolysis inhibitors capable of sensitizing cisplatin-resistant OC cells to therapy holds significant therapeutic potential.

Natural products have been incorporated with therapy for treating various diseases, including cancer, for thousands of years in Asian countries, especially Korea, China, and Japan [14,15]. Increasing studies have suggested that synergizing natural products with conventional chemotherapies can effectively improve chemotherapy responses via multiple molecular mechanisms [16]. Based on the close relationship between cancer metabolic reprogramming and chemoresistance mentioned above, many compounds derived from natural products could reverse chemotherapeutic drug resistance through metabolic regulation. In addition, natural products are considered neoadjuvants for resensitizing chemotherapy from the metabolic perspective, opening up new avenues for overcoming chemotherapy resistance [17]. Isoalantolactone, a sesquiterpene lactone derived from *Inula helenium* L., is known for its diverse biological activities, including anti-inflammatory [18,19] and anticancer properties [20,21]. However, its potential as a glycolysis inhibitor in cisplatin-resistant OC remains largely unexplored. In this study, comprehensive in vitro and in vivo analyses of the effect of isoalantolactone on glycolysis inhibition, sensitization of cisplatin-resistant OC cells to apoptosis, and suppression of tumor growth in cisplatin-resistant xenograft models were performed. These findings highlight the potential of isoalantolactone as a novel therapeutic agent for overcoming cisplatin resistance in OC and provide a basis for further exploration of combination therapies targeting cancer metabolism.

## 2. Results

### 2.1. Isoalantolactone Markedly Inhibits the Growth of A2780^cisR^ and SNU-8^cisR^ OC Cells

**Figure 1:**
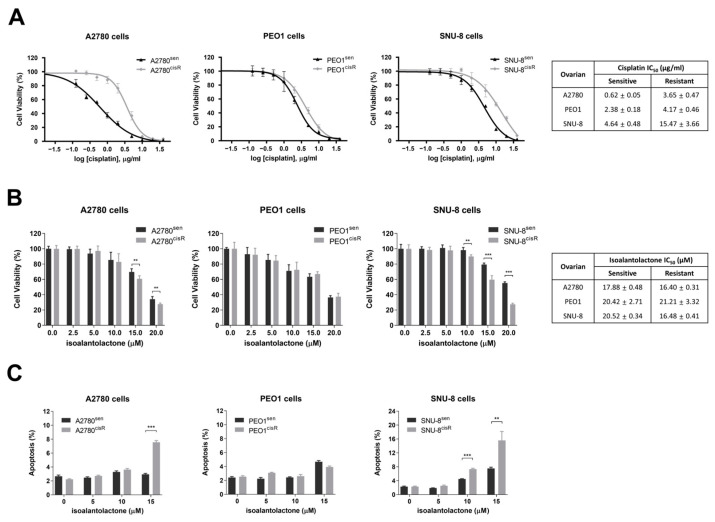
Isoalantolactone potently inhibits the growth of cisplatin-resistant A2780 (A2780^cisR^) and SNU-8 (SNU-8^cisR^) ovarian cancer (OC) cells: (**A**) The resistance phenotype in cisplatin-sensitive and resistant cells of OC A2780, PEO1, and SNU-8 cells. Cells were treated with cisplatin (0–40 µg/mL) and incubated for 48 h. Cell viability was assessed using a cell counting kit-8 assay (CCK-8). The half-maximal inhibitory concentration (IC_50_) values of cisplatin are shown. (**B**) Effect of isoalantolactone on the proliferation of cisplatin-sensitive and resistant OC cells. Cells were treated with isoalantolactone (0–20 µM) for 24 h. CCK-8 assay was performed. IC_50_ values of isoalantolactone are shown. (**C**) Flow cytometric analysis of apoptosis in cisplatin-sensitive and resistant OC cells. Cells were treated with isoalantolactone (0–15 µM) for 48 h. Annexin V staining was performed. Data are representative of three independent experiments as mean ± standard deviation (SD). ** *p* < 0.01; *** *p* < 0.001.

Initially, the significant differences between cisplatin-sensitive and resistant OC cells by using three human OC cell lines (A2780, PEO1, and SNU-8) were evaluated by confirming cell viability. Cisplatin was more cytotoxic to cisplatin-sensitive A2780 cells (A2780^sen^), PEO1 cells (PEO1^sen^), and SNU-8 cells (SNU-8^sen^) than cisplatin-resistant A2780 cells (A2780^cisR^), PEO1 cells (PEO1^cisR^), and SNU-8 cells (SNU-8^cisR^), with a substantial difference between their half-maximal inhibitory concentration (IC_50_) values (Figure 1A), indicating that cisplatin-resistant cells were well established. To determine the effect of isoalantolactone on the proliferation of cisplatin-sensitive and resistant OC cells, OC cells were exposed to different concentrations of isoalantolactone for 24 h. Isoalantolactone was more cytotoxic against A2780^cisR^ and SNU-8^cisR^ than A2780^sen^ and SNU-8^sen^ cells, respectively (Figure 1B). Further evaluation of the effect of isoalantolactone on apoptosis using Annexin V staining indicated that the numbers of apoptotic cells were significantly increased in both A2780^cisR^ and SNU-8^cisR^ compared with A2780^sen^ and SNU-8^sen^ cells, respectively (Figure 1C and Figure S1). However, PEO1^sen^ and PEO1^cisR^ showed no significant differences in cell proliferation and apoptosis. Collectively, these results indicate that isoalantolactone effectively regulates cisplatin-resistant cells, particularly A2780 and SNU-8 OC cells.

### 2.2. Isoalantolactone Inhibits Elevated Glycolysis in A2780^cisR^ and SNU-8^cisR^ OC Cells

**Figure 2:**
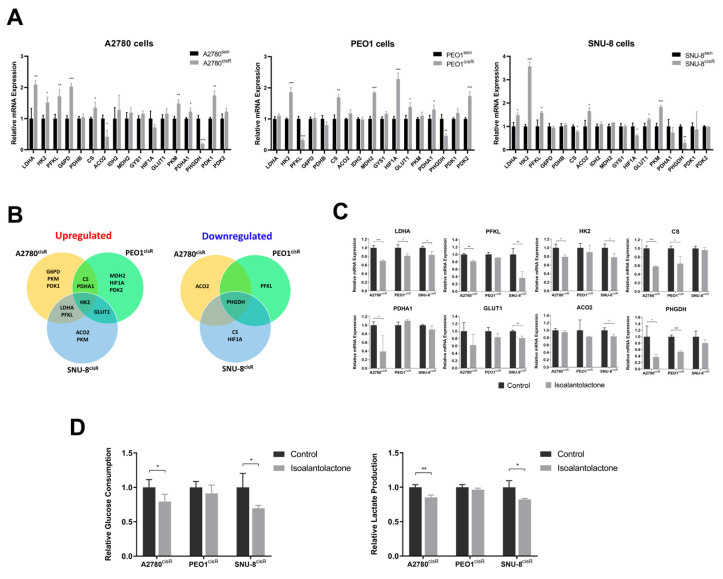
Isoalantolactone alters the elevated glucose metabolism in A2780^cisR^ and SNU-8^cisR^ OC cells: (**A**) Alteration in glycolytic metabolic genes of cisplatin-sensitive and resistant A2780, PEO1, and SNU-8 OC cells. The mRNA level was analyzed via quantitative RT-PCR analysis. (**B**) The upregulated (**left**) and downregulated (**right**) genes in A2780^cisR^, PEO-1^cisR^, and SNU-8^cisR^ OC cells. The overlapping genes between OC cells are indicated in the Venn diagram. (**C**) The effect of isoalantolactone on the mRNA expression of the overlapping genes, *LDHA*, *PFKL*, *HK2*, *CS*, *PDHA1*, *GLUT1*, *ACO2*, and *PHGDH*. (**D**) The effect of isoalantolactone on glucose consumption and lactate production. A2780^cisR^, PEO1^cisR^, and SNU-8^cisR^ OC cells were treated with 10 µM isoalantolactone for 24 h. Glucose consumption and lactate production were measured in the culture media from the cells. The results are presented as mean ± SD (*n* = 4). * *p* < 0.05; ** *p* < 0.01; *** *p* < 0.001. *LDHA*, lactate dehydrogenase A; *HK2*, hexokinase 2; *PFKL*, phosphofructokinase liver type; *G6PD*, glucose 6-phosphate dehydrogenase; *PDHB*, pyruvate dehydrogenase β; *CS*, citrate synthase; *ACO2*, aconitase 2; *IDH2*, isocitrate dehydrogenase 2; *MDH2*, malate dehydrogenase 2; *GYS1*, glycogen synthase 1; *HIF1A*, hypoxia-inducible factor 1α; *GLUT1*, glucose transporter protein type 1; *PKM*, pyruvate kinase M1/2; *PDHA1*, pyruvate dehydrogenase E1α; *PHGDH*, phosphoglycerate dehydrogenase; *PDK1*, pyruvate dehydrogenase kinase 1; *PDK2*, pyruvate dehydrogenase kinase 2.

The alteration in glycolytic metabolic genes in cisplatin-sensitive and resistant OC cells was investigated. A comparative mRNA expression analysis of the two cell types revealed increased glycolysis-related mRNA expression in cisplatin-resistant OC cells (Figure 2A). Specifically, A2780^cisR^ cells exhibited greater mRNA expression levels of lactate dehydrogenase A (*LDHA*), hexokinase 2 (*HK2*), phosphofructokinase liver type (*PFKL*), glucose 6-phosphate dehydrogenase (*G6PD*), citrate synthase (*CS*), pyruvate kinase M1/2 (*PKM*), pyruvate dehydrogenase E1α (*PDHA1*), and pyruvate dehydrogenase kinase 1 (*PDK1*) than A2780^sen^ cells. While PEO1^cisR^ cells demonstrated higher mRNA expression levels of *HK2*, *CS*, malate dehydrogenase 2 (*MDH2*), hypoxia-inducible factor 1α (*HIF1A*), glucose transporter protein type 1 (*GLUT1*), *PDHA1*, and pyruvate dehydrogenase kinase 2 (*PDK2*) than PEO1^sen^ cells, SNU-8^cisR^ cells exhibited greater mRNA expression levels of *LDHA*, *HK2*, *PFKL*, aconitase 2 (*ACO2*), *GLUT1*, and *PKM* than SNU-8^sen^ cells. Given the upregulation of the overlapping candidates *LDHA*, *PFKL*, *CS*, *PDHA1*, *HK2*, and *GLUT1* and the downregulation of phosphoglycerate dehydrogenase (*PHGDH*) in cisplatin-resistant OC cells (Figure 2B), the inhibitory effect of isoalantolactone on the glycolysis of cisplatin-resistant OCs was mainly focused. As shown in Figure 2C, isoalantolactone effectively inhibited glycolysis by targeting key enzymes, including *LDHA* (in A2780^cisR^, PEO1^cisR^, SNU-8^cisR^), *PFKL* and *HK2* (in A2780^cisR^, SNU-8^cisR^), and *CS* and *PHGDH* (in A2780^cisR^, PEO1^cisR^). Furthermore, the evaluation for the determination of whether isoalantolactone induces a metabolic shift in glycolysis in cisplatin-resistant OC cells revealed that isoalantolactone treatment significantly decreased glucose consumption and lactate production in A2780^cisR^ and SNU-8^cisR^ cells (Figure 2D) but failed to do so in PEO1^cisR^ cells. Collectively, these findings suggest that isoalantolactone suppresses glycolysis in cisplatin-resistant OC cells by reducing glycolytic enzymes, leading to decreased glucose consumption and lactate production in cisplatin-resistant OC A2780^cisR^ and SNU-8^cisR^ cells.

### 2.3. Isoalantolactone Increases the Sensitivity of A2780^cisR^ and SNU-8^cisR^ OC Cells to Cisplatin via the Apoptotic Pathway

**Figure 3:**
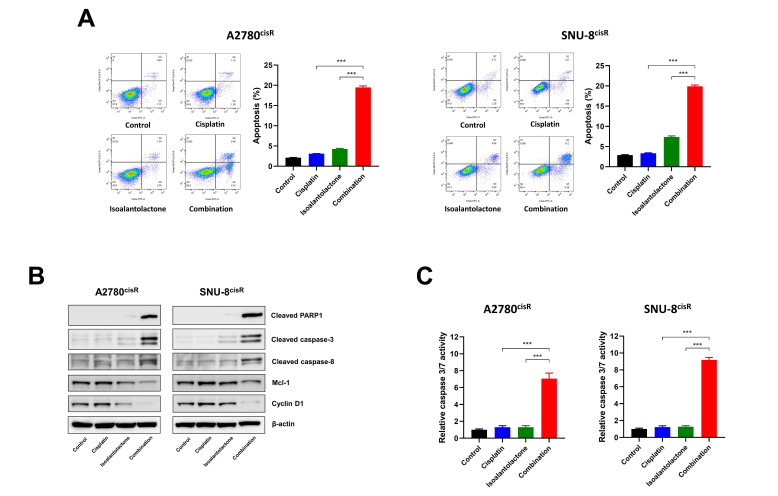
Isoalantolactone sensitizes A2780^cisR^ and SNU-8^cisR^ OC cells to cisplatin via an apoptotic pathway. Cells were treated with 10 µM isoalantolactone combined with cisplatin 2 µg/mL (A2780^cisR^) and 5 µg/mL (SNU-8^cisR^) for 48 h: (**A**) Flow cytometric analysis of apoptosis in A2780^cisR^ and SNU-8^cisR^ OC cells. (**B**) The combined effect of isoalantolactone–cisplatin on the expression of apoptotic proteins. Cell lysates from A2780^cisR^ and SNU-8^cisR^ OC cells were confirmed using Western blotting. (**C**) The combined effect of isoalantolactone–cisplatin on caspase-3/7 activities. The caspase 3/7 activity was evaluated using the Caspase-Glo 3/7 Assay Kit. The results are presented as mean ± SD (*n* = 3). *** *p* < 0.001.

Next, we investigated whether the decreased glycolysis caused by isoalantolactone could affect the sensitivity of cisplatin-resistant OC cells to cisplatin. The results showed that isoalantolactone combined with cisplatin increased apoptosis (Figure 3A and Figure S2), indicating that isoalantolactone treatment sensitized A2780^cisR^ and SNU-8^cisR^ OC cells to cisplatin. The evaluation of the effect of isoalantolactone on apoptosis-related proteins revealed that isoalantolactone increased the expression of cleaved caspase-3, caspase-8, and PARP1 and decreased the expression of anti-apoptotic genes such as cyclin D1 and mcl-1 (Figure 3B). Notably, the Caspase-Glo 3/7 Assay Kit was used to confirm that combination treatment induces apoptosis in OC cells, revealing that isoalantolactone–cisplatin treatment significantly increased caspase-3/7 activities, which agreed with the apoptosis results (Figure 3C). Overall, these results suggest that isoalantolactone suppresses glycolysis and sensitizes cisplatin-resistant cells to cisplatin by inducing mitochondria-mediated apoptosis and caspase activation in A2780^cisR^ and SNU-8^cisR^ cells.

### 2.4. Isoalantolactone Potentiates Cisplatin-Induced Apoptosis by Regulating the Survival Signaling Pathway

**Figure 4:**
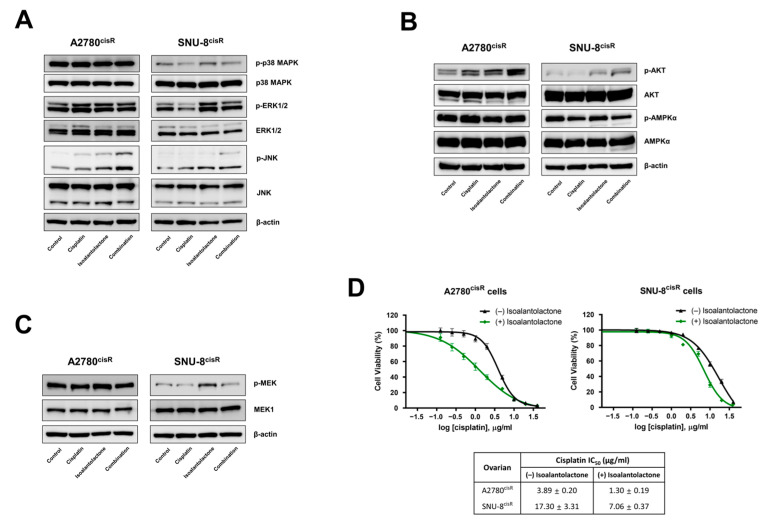
Isoalantolactone combined with cisplatin regulates the survival signaling pathways in A2780^cisR^ and SNU-8^cisR^ OC cells. Cells were treated with cisplatin 2 µg/mL (A2780^cisR^) and 5 µg/mL (SNU-8^cisR^) or in combination with 10 µM isoalantolactone for 24 h: (**A**) The combined effect of isoalantolactone–cisplatin on the MAPK signaling pathway. The phosphorylation of p38 MAPK, ERK1/2, and JNK was confirmed using Western blotting. (**B**) The combined effect of isoalantolactone–cisplatin on AKT and AMPK signaling. The phosphorylation of AKT and AMPK was confirmed using Western blotting. (**C**) The combined effect of isoalantolactone–cisplatin on MEK signaling. Cell lysates from A2780^cisR^ and SNU-8^cisR^ OC cells were confirmed using Western blotting. (**D**) The attenuation of cisplatin resistance by isoalantolactone in A2780^cisR^ and SNU-8^cisR^ OC cells. Cells were treated with 10 μM isoalantolactone, followed by cisplatin (0–40 µg/mL) for 48 h. Cell viability was assessed using CCK-8 assay. IC_50_ values are shown.

AKT, mitogen-activated protein kinase (MAPK), and AMP-activated protein kinase (AMPK) pathways are important for glucose homeostasis in cancer cell survival [22]. To explore the mechanism underlying the effect of isoalantolactone on cisplatin resistance-associated signaling pathways, the protein expression of MAPK signaling pathways was examined. Isoalantolactone–cisplatin treatment resulted in increased phosphorylation of JNK, whereas the total form of JNK remained unchanged in both A2780^cisR^ and SNU-8^cisR^ cells (Figure 4A). Isoalantolactone treatment induced increased phosphorylation of p38 MAPK and ERK1/2, whereas cisplatin treatment decreased levels of these proteins in SNU-8^cisR^ cells. However, they had no effect in A2780^cisR^ cells. In addition, the combination treatment increased AKT phosphorylation in both A2780^cisR^ and SNU-8^cisR^ cells but did not affect AMPKα phosphorylation (Figure 4B). Furthermore, the evaluation of the effect of isoalantolactone treatment on the MEK1 pathway, which is associated with ERK signaling and cisplatin resistance in cancer cells [23], revealed that isoalantolactone increased MEK1 phosphorylation (Figure 4C). The decreased IC_50_ values of cisplatin with isoalantolactone treatment in A2780^cisR^ and SNU-8^cisR^ cells were also confirmed (Figure 4D). Overall, these data suggest that isoalantolactone enhances the cisplatin sensitivity of cisplatin-resistant OC cells by regulating the MAPK and AKT survival signaling pathways.

### 2.5. Isoalantolactone–Cisplatin Treatment Inhibits A2780^cisR^ Xenograft Tumor Growth

**Figure 5:**
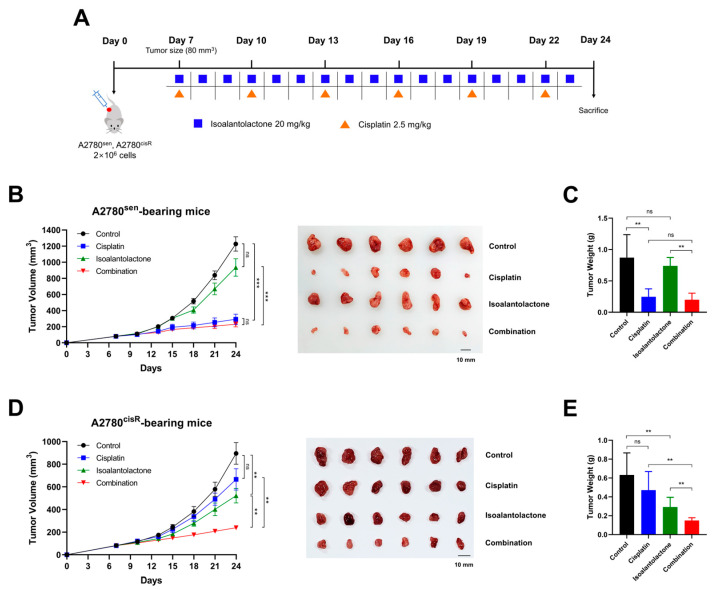
Isoalantolactone combined with cisplatin inhibits A2780^cisR^ xenograft tumor growth: (**A**) Timeline of A2780^sen^ and A2780^cisR^ cell inoculation and treatment with cisplatin and isoalantolactone. A2780^sen^ and A2780^cisR^ cells (2 × 10^6^ cells) were inoculated subcutaneously into Balb/c-nude mice. One week after cell injection, the mice were divided into four groups (*n* = 6, each group) as follows: control, cisplatin, isoalantolactone, and combination (cisplatin–isoalantolactone) groups. Mice were intraperitoneally administered isoalantolactone (20 mg/kg) every day and/or cisplatin (2.5 mg/kg) every three days. Tumor volumes were measured three times a week. (**B**) Time courses of A2780^sen^ cell xenograft tumor growth and visual comparison of dissected A2780^sen^ tumor tissues. (**C**) A2780^sen^ tumor weight. (**D**) Time courses of A2780^cisR^ cell xenograft tumor growth and visual comparison of dissected A2780^cisR^ tumor tissues. (**E**) A2780^cisR^ tumor weight. The results are presented as mean ± standard error of the mean for tumor volume and SD for tumor weight (*n* = 6); ns, not significant; ** *p* < 0.01; *** *p* < 0.001.

To investigate the antitumor effects of isoalantolactone in vivo, A2780^sen^ and A2780^cisR^ cells were used to construct a cisplatin-sensitive and resistant OC model (Figure 5A). In the A2780^sen^ xenograft model, cisplatin treatment dramatically reduced tumor volume (Figure 5B) and tumor weight (Figure 5C) compared with the control group. However, isoalantolactone slightly decreased the tumor volume and tumor weight, although it was not significant. In the A2780^cisR^ xenograft model, isoalantolactone treatment suppressed tumor volume (Figure 5D) and tumor weight (Figure 5E), while cisplatin treatment failed to do so. However, the combination treatment further decreased tumor volume and tumor weight. Isoalantolactone and/or cisplatin treatment did not affect the body weight of the mice compared with the control group, indicating that mouse health remained intact (Figure S3). Overall, these in vivo data suggest that isoalantolactone–cisplatin treatment can effectively overcome cisplatin resistance in OC.

### 2.6. Network Pharmacology Analysis Predicts the Target Pathways of Isoalantolactone-Regulated Glycolysis for OC Treatment

**Figure 6:**
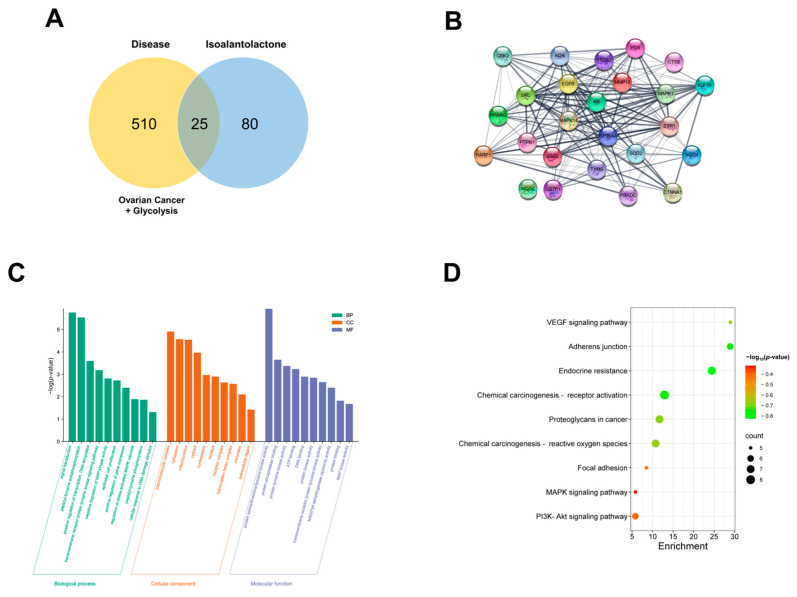
Network pharmacology analysis predicts the target pathways of isoalantolactone-regulated glycolysis for OC treatment: (**A**) Venn diagram of targets between diseases and isoalantolactone. The 535 target genes of the disease were obtained from GeneCards. The 105 potential protein targets for isoalantolactone were obtained from Pharmmapper and SwissTarget. The 25 overlapping gene targets are shown. (**B**) The protein–protein interaction (PPI) network diagram. The common targets of diseases and isoalantolactone were uploaded to the STRING database. *Homo Sapiens* was selected to construct the PPI network. (**C**) Gene Ontology (GO) functional enrichment analyses. Green represents biological process (BP), orange represents cellular component (CC), and purple represents molecular function (MF) terms for GO analysis. (**D**) The Kyoto Encyclopedia of Genes and Genomes pathway enrichment analysis of potential core targets.

Network pharmacology provides a systemic approach to unraveling the potential multiple actions of natural product-derived compounds [24]. Information regarding the 105 potential protein targets for isoalantolactone was obtained using Pharmmapper and SwissTarget. Information regarding the 535 target genes of the disease was obtained using GeneCards. The 25 common gene targets are shown in Figure 6A. Potential targets were submitted to the STRING database, and the protein–protein interaction (PPI) network was constructed. The predicted PPI network showed that a functional link may exist between those target genes of isoalantolactone (Figure 6B). Furthermore, Gene Ontology (GO) and the Kyoto Encyclopedia of Genes and Genomes (KEGG) pathway enrichment analyses were performed. As shown in Figure 6C, the biological process terms for GO analysis included signal transduction, MAPK activity, and phosphorylation. The cellular component terms for GO analysis included macromolecular complex, cytoplasm, and mitochondrion. The molecular function terms for GO analysis included protein kinase activity, protein phosphatase binding, and MAPK activity. In addition, the KEGG pathway enrichment analysis of potential core targets indicated that genes might influence the VEGF, MAPK, and AKT pathways (Figure 6D). These results indicate that isoalantolactone may act on OC-related genes and that the MAPK pathway may be a potential treatment pathway.

## 3. Discussion

The mechanism of cisplatin resistance is extremely complex and multifactorial. One of the key mechanisms underlying cisplatin resistance in cancer is dysregulated cellular metabolism, particularly upregulated glycolysis [25]. Glucose is mainly used in OC cells to produce ATP and to maintain redox homeostasis and energy balance. Previous studies demonstrated that cisplatin-resistant OC cells require a higher demand for glucose and are more sensitive to glucose deprivation [26,27]. Similarly, in this study, cisplatin-resistant OC cells exhibited an enhanced glycolytic phenotype, proven by the upregulation of glycolytic enzymes such as *LDHA*, *PFKL*, and *HK2*. Among these enzymes, *HK2*-encoding gene is commonly upregulated in cisplatin-resistant cell lines used in experiments. *HK2* phosphorylates glucose to generate glucose-6-phosphate, the first step in glycolysis [28]. A high expression of *HK2* in tumors promotes tumor growth by maintaining higher glycolysis rates in cancer cells. In addition, *HK2* overexpression has been correlated to chemoresistance in tumors [29]. Interestingly, cisplatin treatment reduced *HK2* expression in cisplatin-sensitive OC cells but not in cisplatin-resistant OC cells [30]. *HK2*-targeting inhibitors combined with cisplatin could represent a novel approach to overcome cisplatin resistance. *LDHA* controls the conversion of pyruvate to lactate. Inhibiting glycolysis by targeting *LDHA* could restore cisplatin sensitivity in cisplatin-resistant cells [31]. Targeting *PFKL*, a rate-limiting glycolysis enzyme, could reduce glucose consumption and lactate production [32]. Therefore, small molecules targeting glycolytic enzymes have recently gained more attention in chemoresistance.

Natural products are a potential source of drug discovery. Small molecules derived from natural products can exhibit various pharmacological properties, including anticancer activities [33]. Although isoalantolactone exhibits anticancer activities in several types of cancer cells [34,35,36,37], including imatinib-resistant chronic myeloid leukemia [38], its anticancer effect in the cisplatin-resistant model and whether it can regulate cancer metabolism remain largely unknown. In particular, cisplatin resistance remains a significant challenge in OC treatment, necessitating the exploration of novel therapeutic approaches [39,40]. In this study, the potential of isoalantolactone as a glycolysis inhibitor was investigated to overcome cisplatin resistance in OC. The results demonstrated that isoalantolactone effectively inhibited the growth of cisplatin-resistant OC cells, particularly A2780^cisR^ and SNU-8^cisR^ cells. These findings suggest that isoalantolactone exhibits stronger cytotoxicity toward cisplatin-resistant OC cells than cisplatin-sensitive cells, highlighting its potential as a promising therapeutic agent. In addition, isoalantolactone effectively targeted these key enzymes, resulting in significantly inhibited glycolysis in cisplatin-resistant OC A2780^cisR^ and SNU-8^cisR^ cells. It reduced glucose consumption and lactate production, suggesting the disruption of the glycolytic pathway. These findings highlight the potential of isoalantolactone as a glycolysis inhibitor in cisplatin-resistant OC cells.

While the upregulation of glycolysis plays a crucial role in enabling cancer cells to evade cisplatin-induced apoptosis [41,42], its inhibition can enhance the chemotherapeutic sensitivity of OC to cisplatin [43]. Importantly, isoalantolactone sensitized cisplatin-resistant OC cells to cisplatin-induced apoptosis, and isoalantolactone–cisplatin treatment resulted in increased apoptosis compared with the individual treatments. This suggests that isoalantolactone inhibits glycolysis and enhances the efficacy of cisplatin by promoting apoptotic cell death in cisplatin-resistant OC cells. Many signaling pathways are associated with cisplatin resistance, as they promote cell survival and proliferation [44]. The activation of MAPK and AKT signaling pathways in cisplatin resistance has been demonstrated [45,46]. Isoalantolactone–cisplatin treatment was associated with the inhibition of survival signaling pathways, as indicated by the increased phosphorylation of JNK and AKT. These findings suggest that isoalantolactone overcomes cisplatin resistance by modulating survival signaling pathways and inducing apoptosis. Although isoalantolactone has a therapeutic effect on the recovery of chemoresistance through alteration in glucose metabolism and survival signaling pathways, further studies are required to clarify their relationship and the exact mechanism of isoalantolactone.

In this study, in vivo analyses using cisplatin-sensitive and resistant OC xenograft models were conducted to clarify the potential of isoalantolactone in overcoming cisplatin resistance. While cisplatin treatment alone showed limited efficacy in reducing tumor growth in the cisplatin-resistant model, isoalantolactone treatment significantly suppressed tumor growth. Surprisingly, isoalantolactone–cisplatin treatment further enhanced the antitumor effect, indicating a synergistic interaction between these treatments. These results suggest that isoalantolactone, either alone or in combination with cisplatin, can effectively overcome cisplatin resistance in OC. In addition, a network pharmacology analysis revealed the potential targets and pathways associated with isoalantolactone-regulated glycolysis for OC treatment. The analysis identified several key pathways, including the VEGF, MAPK, and AKT pathways, which have been implicated in cancer progression and drug resistance [47,48]. These findings provide a theoretical basis for the therapeutic potential of isoalantolactone in OC and suggest the involvement of specific pathways that may be targeted by isoalantolactone to overcome cisplatin resistance.

## 4. Materials and Methods

### 4.1. Chemicals and Reagents

Gibco (Grand Island, NY, USA) supplied RPMI-1640 medium, fetal bovine serum (FBS), and Dulbecco’s phosphate-buffered saline (DPBS). ChemFaces (Wuhan, China) supplied isoalantolactone. Antibodies against cleaved PARP1, cleaved caspase-3, cleaved caspase-8, cyclin D1, mcl-1, p-p38 MAPK, p38 MAPK, p-ERK1/2, ERK1/2, p-AKT, AKT, p-AMPKα, AMPKα, p-MEK, MEK, and β-actin were purchased from Cell Signaling Technology (Beverly, MA, USA). Santa Cruz Biotechnology (Santa Cruz, CA, USA) supplied antibodies against p-JNK and JNK. Cisplatin, dimethyl sulfoxide (DMSO), and Tween 80 were purchased from Sigma-Aldrich (St. Louis, MO, USA).

### 4.2. Cell Culture

The European Collection of Authenticated Cell Cultures supplied A2780^sen^, PEO1^sen^, A2780^cisR^, and PEO1^cisR^ OC cells. SNU-8^sen^ OC cells were purchased from the Korean Cell Line Bank. SNU-8^cisR^ OC cells were established via continuous exposure to increasing doses of cisplatin over 1 year. The cells were maintained in an RPMI-1640 medium containing 10% FBS, penicillin 100 U/mL, and streptomycin 100 μg/mL at 37 °C in a 5% CO_2_ humidified atmosphere.

### 4.3. Cell Viability Assay

Cell proliferation was determined using a cell counting kit-8 assay (CCK-8, Dojindo, Kumamoto, Japan). Briefly, cells at the density of 1 × 10^4^ cells/well (A2780 and PEO1) and 5 × 10^3^ cells/well (SNU-8) were seeded into 96-well plates and incubated overnight. Then, different concentrations of cisplatin and/or isoalantolactone were added to each well. After 24 h or 48 h, CCK-8 was added. The absorbance of the CCK-8 reagent was detected at 450 nm using a Spectramax i3 microplate reader (Molecular Devices, San Jose, CA, USA). IC_50_ of cisplatin was calculated using GraphPad Prism 9 (San Diego, CA, USA).

### 4.4. Apoptosis Analysis

A flow cytometric analysis of apoptosis was performed to evaluate the apoptosis-inducing effects of isoalantolactone. Here, 5 × 10^5^ of A2780^cisR^ cells and 2.5 × 10^5^ of SNU-8^cisR^ were seeded into 6-well plates and maintained in a 37 °C incubator overnight. Afterward, the cells were exposed to a medium containing isoalantolactone (10 μM), followed by cisplatin (2 μg/mL for A2780^cisR^ cells and 5 μg/mL for SNU-8^cisR^ cells) for 48 h. The cells were detached, washed in DBPS, and resuspended in Annexin V binding buffer, followed by the addition of Annexin V/FITC and propidium iodide and 15 min incubation in the dark. The cells were resuspended in 400 μL of Annexin V binding buffer. The stained cells were acquired and analyzed using a BD LSRFortessa™ X-20 flow cytometer (BD Biosciences, San Jose, CA, USA) and FlowJo 10.8 (BD Biosciences).

### 4.5. Quantitative RT-PCR

Total RNA from A2780, PEO1, and SNU-8 cells was isolated using an RNeasy Plus Mini Kit (Qiagen, Valencia, CA, USA). The purity and concentration of RNA were measured using NanoDrop (Thermo Scientific, Waltham, MA, USA). Total RNA (1 μg) was converted to cDNA using iScript™ Advanced cDNA Synthesis Kit (Bio-Rad, Hercules, CA, USA). Table 1 provides the primer sequences. Gene expression analysis was performed using the SsoAdvanced SYBR Green SuperMix Kit (Bio-Rad) on the Bio-Rad CFX Connect Real-Time System (Bio-Rad). The relative gene level was calculated using comparative delta Ct and normalized using the *ACTB* gene.

### 4.6. Metabolic Assays

Alteration in cellular metabolism regarding glycolysis was measured using the Glucose-Glo ™ Assay and Lactate-Glo ™ Assay (Promega, Madison, WI, USA). The cells at densities of 1 × 10^4^/well (A2780^cisR^ cells) and 5 × 10^3^/well (SNU-8^cisR^ cells) were seeded in 96-well plates and incubated overnight. The cells were treated with isoalantolactone (10 μM), followed by cisplatin (2 μg/mL for A2780^cisR^ cells and 5 μg/mL for SNU-8^cisR^ cells) for 24 h. The culture and control media were collected and incubated with glucose detection reagent and lactate detection reagent for 1 h, respectively. The luminescence intensity was measured using a microplate luminometer (Molecular Devices). Glucose consumption and lactate production were calculated by subtracting from the concentrations of glucose and lactate in the control medium.

### 4.7. Western Blotting

Cell lysates were prepared using NP40 Cell Lysis Buffer (Invitrogen, Carlsbad, CA, USA), supplemented with Halt™ Protease and Phosphatase Inhibitor Cocktail (Thermo Fisher Scientific, Waltham, MA, USA). Equal amounts of protein, determined with a Qubit™ Protein Assay Kit (Invitrogen), were prepared in 4× LDS sample buffer (Invitrogen) and 10× sample reducing agent (Invitrogen) and heated at 70 °C for 10 min. The samples were separated on Bolt 4 to 12% Bis-Tris gels in Bolt MOPS SDS running buffer (Thermo Fisher Scientific), and the proteins were transferred onto a nitrocellulose membrane (Invitrogen) using the SureLock Tandem Transfer System (Thermo Fisher Scientific). The membrane was blocked with EveryBlot Blocking Buffer (Bio-Rad), incubated with primary antibodies overnight, and incubated with secondary antibodies conjugated with horseradish peroxidase. The bands were developed using Clarity ECL Western Blotting Substrates (Bio-Rad) and visualized using an ImageQuant LAS 4000 mini (GE Healthcare, Chicago, IL, USA).

### 4.8. Measurement of Caspase-3/7 Activity

The caspase-3/7 activities were evaluated using a Caspase-Glo 3/7 Assay Kit (Promega) following the manufacturer’s instructions. Briefly, cells at densities of 5 × 10^3^ cells/well (A2780) and 2.5 × 10^3^ cells/well (SNU-8) were cultured in 96-well plates and incubated overnight, followed by exposure to a medium containing isoalantolactone (10 μM) and subsequently cisplatin (2 μg/mL for A2780^cisR^ cells and 5 μg/mL for SNU-8^cisR^ cells) for 48 h. Caspase-3/7 reagent was added to each well and incubated for 1 h at room temperature. The luminescence intensity was measured using a microplate luminometer (Molecular Devices).

### 4.9. Tumor-Bearing Mice and Treatment

Specific pathogen-free five-week-old female Balb/c-nude mice were purchased from Saeron Bio (Uiwang, Republic of Korea) and acclimated for one week prior to experimental use. The mice were housed under the following environmental conditions: temperature 23 °C; humidity 50%; 12 h light–dark cycle. The animals were fed a standard chow diet (Purina Co., Seoul, Republic of Korea) and given free access to drinking water. A2780^sen^ and A2780^cisR^ tumor-bearing mice were prepared by being injected subcutaneously with 2 × 10^6^ cells into the right flank. After the tumor challenge, Balb/c-nude mice were intraperitoneally injected with 20 mg/kg isoalantolactone every day and 2.5 mg/kg cisplatin every three days for 17 days. Cisplatin was dissolved in PBS. Isoalantolactone was dissolved in PBS containing 2% DMSO and 2% Tween 80. All experimental procedures were approved by the Animal Care and Use Committee of the Korea Institute of Oriental Medicine (Approval Number: 21-032). Tumor volume was measured via caliper measurement and calculated using the following formula: length × width^2^ × 1/2. Body weight and tumor size were recorded.

### 4.10. Systematic Pharmacological Analysis of Isoalantolactone

Using GeneCards (https://genecards.weizmann.ac.il/v3/, accessed on 25 May 2023), the target genes of the disease were acquired as the keyword of “ovarian cancer” and “glycolysis”. The PubChem (https://pubchem.ncbi.nlm.nih.gov/, accessed on 17 October 2022) database was used to obtain the structure of isoalantolactone. Pharmmapper (https://www.lilab-ecust.cn/pharmmapper/index.html, accessed on 17 October 2022) and SwissTarget (https://www.swisstargetprediction.ch/, accessed on 17 October 2022) databases with the “Homo sapiens” species setting were utilized to predict the protein targets of isoalantolactone. The predicted protein targets were converted into the predicted gene targets using the UniProt database (https://www.uniport.org/, accessed on 25 May 2023). The disease–gene targets and drug-prediction gene targets were combined through Venny 2.1 software (https://bioinfogp.cnb.csic.es/tools/venny/index.html, accessed on 25 May 2023) to obtain common gene targets. The STRING database (https://cn.string-db.org/, accessed on 1 June 2023) was used to construct a protein–protein interaction network. GO and KEGG pathway enrichment analyses were performed on the David online analysis platform (https://david.ncifcrf.gov/, accessed on 2 June 2023) to screen the GO processes and signaling pathways involved in the potential common targets of isoalantolactone and diseases. The bubble chart and bar graph were plotted using an online platform (https://www.bioinformatics.com.cn/, accessed on 2 June 2023) for visualization.

### 4.11. Statistical Analysis

Statistical analysis was performed using GraphPad Prism 9.0. Error bars represent mean ± standard deviation (SD), except for tumor volume curves, which represent mean ± standard error of the mean. Statistical analysis of significance was performed using a two-tailed Student’s *t-*test. A value of *p* < 0.05 was considered statistically significant.

## 5. Conclusions

In conclusion, this study demonstrates the efficacy of isoalantolactone as a glycolysis inhibitor in overcoming cisplatin resistance in OC. Isoalantolactone effectively targets glycolytic enzymes and suppresses glycolysis. In addition, isoalantolactone supports the action of anticancer drugs by increasing the sensitivity of OC cells to cisplatin and helping to overcome cisplatin resistance. These findings highlight the potential of isoalantolactone as a novel therapeutic strategy for overcoming cisplatin resistance in OC. Further studies are warranted to elucidate the precise molecular mechanisms underlying its action and to evaluate its clinical potential.

## Figures and Tables

**Table 1:** List of primer sequences for quantitative RT-PCR.

Target Genes	Forward Sequences	Reverse Sequences
*LDHA*	GGA TCT CCA ACA TGG CAG CCT T	AGA CGG CTT TCT CCC TCT TGC T
*HK2*	GAG TTT GAC CTG GAT GTG GTT GC	CCT CCA TGT AGC AGG CAT TGC T
*PFKL*	AAG AAG TAG GCT GGC ACG ACG T	GCG GAT GTT CTC CAC AAT GGA C
*G6PD*	CTG TTC CGT GAG GAC CAG ATC T	TGA AGG TGA GGA TAA CGC AGG C
*PDHB*	TGT AAC TGT GGA AGG AGG CTG G	CAT CAG CAC CAG TGA CAC GAA C
*CS*	CAC AGG GTA TCA GCC GAA CCA A	CCA ATA CCG CTG CCT TCT CTG T
*ACO2*	CAA TCG TCA CCT CCT ACA ACA GG	GTC TCT GGG TTG AAC TTG AGG G
*IDH2*	AGA TGG CAG TGG TGT CAA GGA G	CTG GAT GGC ATA CTG GAA GCA G
*MDH2*	CTG GAC ATC GTC AGA GCC AAC A	GGA TGA TGG TCT TCC CAG CAT G
*GYS1*	CCG CTA TGA GTT CTC CAA CAA GG	AGA AGG CAA CCA CTG TCT GCT C
*HIF1A*	TAT GAG CCA GAA GAA CTT TTA GGC	CAC CTC TTT TGG CAA GCA TCC TG
*GLUT1*	TTG CAG GCT TCT CCA ACT GGA C	CAG AAC CAG GAG CAC AGT GAA G
*PKM*	ATG GCT GAC ACA TTC CTG GAG C	CCT TCA ACG TCT CCA CTG ATC G
*PDHA1*	GGA TGG TGA ACA GCA ATC TTG CC	TCG CTG GAG TAG ATG TGG TAG C
*PHGDH*	CTT ACC AGT GCC TTC TCT CCA C	GCT TAG GCA GTT CCC AGC ATT C
*PDK1*	CAT GTC ACG CTG GGT AAT GAG G	CTC AAC ACG AGG TCT TGG TGC A
*PDK2*	TGC CTA CGA CAT GGC TAA GCT C	GAC GTA GAC CAT GTG AAT CGG C
*ACTB*	CAC CAT TGG CAA TGA GCG GTT C	AGG TCT TTG CGG ATG TCC ACG T

*LDHA*, lactate dehydrogenase A; *HK2*, hexokinase 2; *PFKL*, phosphofructokinase liver type; *G6PD*, glucose 6-phosphate dehydrogenase; *PDHB*, pyruvate dehydrogenase β; *CS*, citrate synthase; *ACO2*, aconitase 2; *IDH2*, isocitrate dehydrogenase 2; *MDH2*, malate dehydrogenase 2; *GYS1*, glycogen synthase 1; *HIF1A*, hypoxia-inducible factor 1α; *GLUT1*, glucose transporter protein type 1; *PKM*, pyruvate kinase M1/2; *PDHA1*, pyruvate dehydrogenase E1α; *PHGDH*, phosphoglycerate dehydrogenase; *PDK1*, pyruvate dehydrogenase kinase 1; *PDK2*, pyruvate dehydrogenase kinase 2; *ACTB*, β-actin.

## Data Availability

The data presented in this study are available on request from the corresponding author.

## Supplementary Materials

The following supporting information can be downloaded at: https://www.mdpi.com/article/10.3390/ijms241512397/s1.
