# Supplementary material for: Isoalantolactone Suppresses Glycolysis and Resensitizes Cisplatin-Based Chemotherapy in Cisplatin-Resistant Ovarian Cancer Cells
Source: Int J Mol Sci. 2023 Aug 3;24(15):12397. doi: 10.3390/ijms241512397 (PMC10419319; doi:10.3390/ijms241512397)
Supplement: Supplementary file 1 [file ijms-24-12397-s001.zip › ijms-2520410-supplementary.pdf]

International Journal of *Molecular Sciences*

Supplementary Materials

Isoalantolactone Suppresses Glycolysis and Resensitizes Cisplatin-Based Chemotherapy in Cisplatin-Resistant Ovarian Cancer Cells

Jaemoo Chun

KM Convergence Research Division, Korea Institute of Oriental Medicine, Daejeon 34054, Republic of Korea; jchun@kiom.re.kr; Tel.: +82-42-868-9511

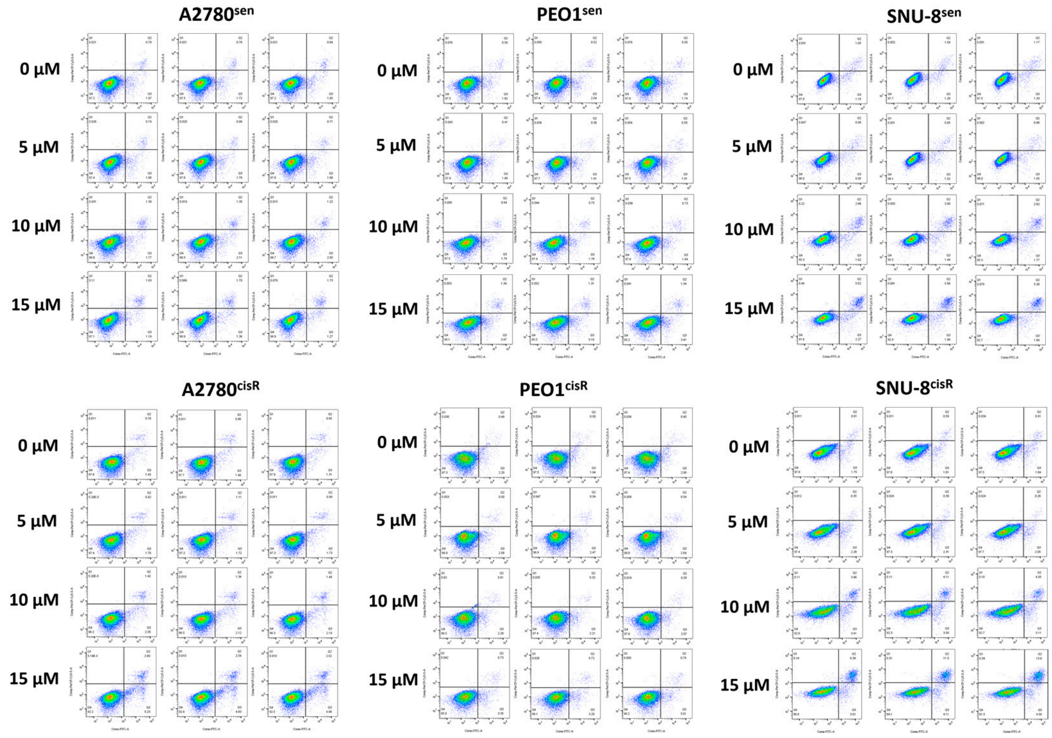

Figure S1. Flow cytometeric analysis of apoptosis in cisplatin-sensitive and resistant ovarian cancer A2780, PEO-1, and SNU-8 cells. The cells were treated with isoalantolactone (0–15 μM) for 48 h. Annexin V staining was performed.

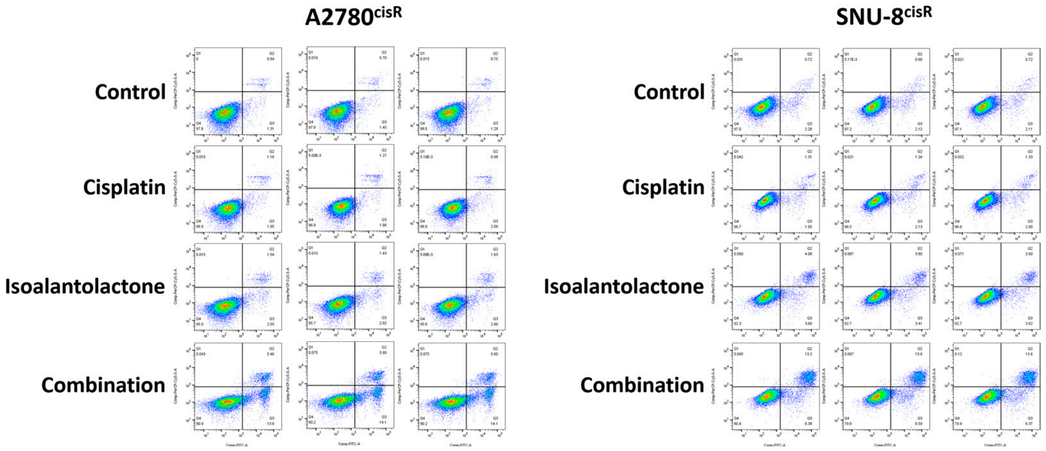

Figure S2. Flow cytometeric analysis of apoptosis in A2780^{cisR} and SNU-8^{cisR} OC cells. The cells were treated with 10 μM isoalantolactone combined with cisplatin 2 μg/ml (A2780^{cisR}) and 5 μg/ml (SNU-8^{cisR}) for 48 h. Annexin V staining was performed.

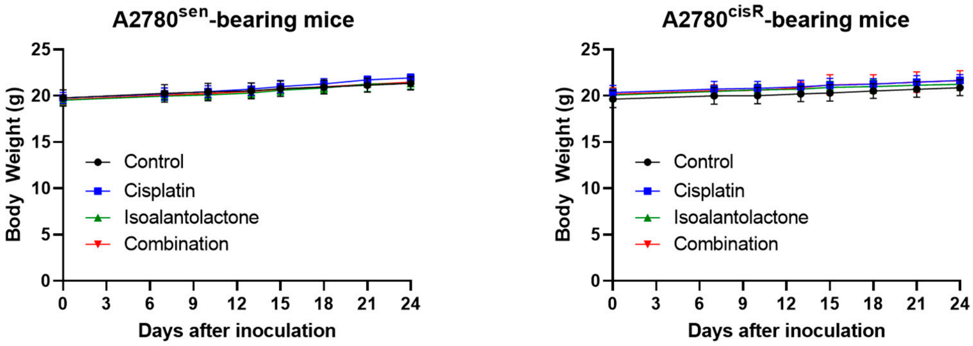

Figure S3. Time courses of animal body weight in A2780^{sen} and A2780^{cisR} xenograft models.
